# Strategies for Detection of *Plasmodium* *species* Gametocytes

**DOI:** 10.1371/journal.pone.0076316

**Published:** 2013-09-27

**Authors:** Rahel Wampfler, Felistas Mwingira, Sarah Javati, Leanne Robinson, Inoni Betuela, Peter Siba, Hans-Peter Beck, Ivo Mueller, Ingrid Felger

**Affiliations:** 1 Swiss Tropical and Public Health Institute, Basel, Switzerland; 2 University of Basel, Basel, Switzerland; 3 Papua New Guinea Institute of Medical Research, Goroka, Papua New Guinea; 4 Infection and Immunity Division, Walter and Eliza Hall Institute, Parkville, Victoria, Australia; Institut Pasteur, France

## Abstract

Carriage and density of gametocytes, the transmission stages of malaria parasites, are determined for predicting the infectiousness of humans to mosquitoes. This measure is used for evaluating interventions that aim at reducing malaria transmission. Gametocytes need to be detected by amplification of stage-specific transcripts, which requires RNA-preserving blood sampling. For simultaneous, highly sensitive quantification of both, blood stages and gametocytes, we have compared and optimized different strategies for field and laboratory procedures in a cross sectional survey in 315 5-9 yr old children from Papua New Guinea. qRT-PCR was performed for gametocyte markers pfs25 and pvs25, *Plasmodium* species prevalence was determined by targeting both, 18S rRNA genes and transcripts. RNA-based parasite detection resulted in a *P. falciparum* positivity of 24.1%; of these 40.8% carried gametocytes. *P. vivax* positivity was 38.4%, with 38.0% of these carrying gametocytes. Sensitivity of DNA-based parasite detection was substantially lower with 14.1% for *P. falciparum* and 19.6% for *P. vivax*. Using the lower DNA-based prevalence of asexual stages as a denominator increased the percentage of gametocyte-positive infections to 59.1% for *P. falciparum* and 52.4% for *P. vivax*. For studies requiring highly sensitive and simultaneous quantification of sexual and asexual parasite stages, 18S rRNA transcript-based detection saves efforts and costs. RNA-based positivity is considerably higher than other methods. On the other hand, DNA-based parasite quantification is robust and permits comparison with other globally generated molecular prevalence data. Molecular monitoring of low density asexual and sexual parasitaemia will support the evaluation of effects of up-scaled antimalarial intervention programs and can also inform about small scale spatial variability in transmission intensity.

## Introduction

The importance of molecular monitoring of gametocytes of *Plasmodium* parasites is increasingly acknowledged, because it provides fast and sensitive quantification of the parasite stage required for transmission from humans to mosquito vectors. Molecular techniques detect particularly very low gametocyte densities that escape detection by light microscopy (LM). Gametocyte densities are an important measure for evaluating effects of interventions that aim at reducing transmission, such as specific drugs, vaccines or bednets [1–3]. Monitoring gametocytes in population studies can inform about the human infective reservoir and provides relevant data for transmission models.

Mature stage V gametocytes circulate in the peripheral blood of infected humans for a mean period of 6.4 days or a maximum of 3 weeks, but often at sub-microscopic levels [2,4]. The proportion of gametocytes among total parasites per host ranged from 0.2% in young children to 5.7% in adults [5]. By molecular techniques gametocytes are differentiated from concurrent asexual forms by targeting RNA transcripts of gametocyte-specifically expressed genes. In the past, detection of submicroscopic gametocytaemia of *P. falciparum* and *P. vivax* was achieved by two non-quantitative methods, reverse transcription-PCR (RT-PCR) and nucleic acid sequence based amplification techniques (NASBA) [4,6–9]. Currently quantitative NASBA techniques are applied increasingly for gametocyte detection of both, *P. falciparum* [10] and *P. vivax* [11]. For this work we have developed and validated quantitative qRT-PCR TaqMan probe-based assays for *P. falciparum* and *P. vivax* gametocytes.

In view of large field studies planned to monitor effects of antimalarial interventions, robust sampling strategies and laboratory assays are needed. For the detection of gametocytes of malaria parasites, we have evaluated and compared several approaches adapted specifically to those meso- to highly endemic settings where several *Plasmodium* species occur together. Diagnosis of multiple species incurs substantial costs for cross sectional surveys or surveillance, when mostly uninfected individuals or asymptomatic parasite carriers require testing. This issue was addressed by introducing a generic screening assay to determine initially in a single experiment all those samples positive for any *Plasmodium* species. Only samples positive in the generic test were carried forward to species-specific and gametocyte assays. Our aim was to devise a parsimonious but sensitive diagnostic approach, generating robust information on asexual stage and gametocyte densities. These strategies should be useful for investigating transmission dynamics in longitudinal studies.

Prompted by previous reports of successful usage of finger prick blood collected on various filter paper brands in the field [6,7], we have compared the efficiency of sampling and storage on filter paper versus in solution. Three different sampling strategies were applied in the field: (i) whole blood stored in RNAprotect® cell reagent, (ii) whole blood spotted onto Whatman® 3MM filter paper, air dried and stored in TRIzol® reagent thereafter, and (iii) Whatman FTA classic cards. The focus of this study was on the practical field work in the endemic settings with realistic time periods and limited access to freezers. This adds to some recent comparisons of laboratory cultured gametocytes under a variety of controlled conditions and stored for a maximum period of 3 months until processing of samples [8,12]. Both these studies provided a good overview on various brands of filter papers. However, under the specific conditions of malaria surveillance or intervention programs, time intervals from sample collection to processing in the molecular laboratory will likely extend beyond 3 months. Therefore we have investigated the stability of RNA in stored blood samples and that of extracted RNA over a two year interval.

High throughput of samples requiring RNA extraction for gametocyte detection becomes a technical challenge in the context of intensified malaria surveillance. RNA extraction from filter papers is more tedious and contamination prone than handling liquid samples in 96-well format. We have therefore investigated the field applicability of RNAprotect solution (Qiagen) which stabilizes RNA for short term storage and transport at ambient temperature and permits RNA extraction in 96 well plates. We made use of the availability of DNA and RNA of each sample for evaluating the diagnostic sensitivity of DNA-based versus RNA-based parasite detection in field samples. Due to the high abundance of transcripts of our molecular marker 18S rRNA we expected detection of very low density infections, even below the detection limit of standard PCR.

In Papua New Guinea (PNG) all four major *Plasmodium* spp. infecting humans co-occur, whereby *P. falciparum* and *P. vivax* are the predominant *Plasmodium* species with similar frequency [13]. Malaria endemicity is geographically variable throughout PNG with variations not only along broad environmental gradients, but also between villages only a few kilometers apart [14] and even between different clusters of houses within the same villages [15,16]. In this work we have compared several sampling methods and molecular diagnostic approaches, the summary of which lead us to propose a parsimonious strategy for high throughput molecular monitoring in endemic areas with several sympatric *Plasmodium* species.

## Materials and Methods

### Study population and ethics

Samples were collected from February to March 2010 from 315 mostly asymptomatic children aged 5 to 9 years. This cross-sectional survey formed part of a major cohort study conducted in the Albinama region of Maprik District, East Sepik Province, a malaria endemic area in PNG. Written informed consent was obtained from parents or guardians of each child. Ethical clearance for all molecular analyses was obtained from the Medical Research Advisory Committee of the PNG Ministry of Health (MRAC no. 1206) and from the Ethics Committee of Basel (no. 237/11).

### Blood collection and sample storage and transport

From each study participant approximately 250µl of blood was collected in the village by finger prick into a BD microtainer™ containing EDTA. Samples were stored by three different methods: (i) 50 µl whole blood spotted on Whatman® 3MM filter paper directly after bleeding, air dried and stored for 2-4 weeks at +4 °C. Then each blood spot was cut into multiple pieces, transferred into a microfuge tube containing 300 µl TRIzol® reagent (Life Technologies, Zug, Switzerland) and stored at -80°C until shipment on wet ice packs to the molecular laboratory; upon arrival filter papers again were stored at -80°C until RNA extraction. When handling filter papers, RNase was eliminated by RNase away™ Reagent (Ambion) (ii). 50 µl whole blood spotted on FTA classic cards (Whatman, cat. number: WB120205) directly after bleeding, air-dried completely, stored at +4°C and shipped with desiccant at ambient temperature, then again stored at -20°C until RNA extraction (iii). After transport to the field laboratory and within a maximum of 4 hours following blood collection, 50 µl whole blood was transferred from the microtainer into 250 µl RNAprotect® cell reagent (Qiagen). The mixture was stored at -20°C until transported with wet ice pack cooling to the molecular laboratory. RNAprotect and FTA card samples were stored for 5-8 months before nucleic acids were extracted. Filter papers in TRIzol were stored for up to 1 year.

The sampling methods described should not be considered optimized procedures, but rather reflect the best possible option under the specific field conditions. Clearly, the shorter the storage at ambient temperature and the time span to RNA extraction, the better. But stricter protocols are often only realizable under trial conditions or when working with in vitro cultured parasites or blood from travel clinics.

### Extraction of nucleic acids

#### RNA extraction

All three blood samples collected from 315 study participants were subject to different RNA extraction protocols that had been optimized with *P. falciparum* 3D7 in vitro culture.

(i) Whatman 3MM filter paper: RNA was extracted from whole blood spotted on Whatman 3MM filter paper (corresponding to 25µl whole blood) and stored in TRIzol. Filter papers were transferred from TRIzol into 600 µl RLT lysis buffer containing β-mercaptoethanol (Qiagen RNeasy® plus mini kit) and incubated for 15 min at 30°C on a shaker at 1000 rpm. After centrifugation for 30 sec at 13000g the aqueous phase was transferred to a gDNA eliminator column, a kit component. From this step onwards, the instructions of the kit supplier (Qiagen) were followed closely. The now following procedure included an on-column DNase digest performed after the first washing step with buffer RW1. 10 µl RNase-free DNase (Qiagen) was mixed with 70 µl RDD buffer and added to the membrane. DNA digestion was allowed to proceed for 15 min at room temperature and then terminated by a wash step with buffer RW1 and the supplier’s protocol was continued. Finally RNA was eluted in 50 µl RNase-free dH2O and stored for short term at -20°C, for long term at -80°C.
(ii) Whatman® FTA classic cards: RNA was extracted from FTA cards using the Qiagen RNeasy® plus mini kit protocol to 5 filter discs punched out from FTA cards impregnated with whole blood using a Harris Micro-Punch (tip diameter 3.0 mm). Discs were vortexed in buffer RLT Plus for 1 minute and the incubated for 30 min at room temperature (24-27°C) followed by a gDNA eliminator column and an on-column DNase digestion (all Qiagen) according to the manufacturer’s protocols. Yields at room temperature were higher than those at incubation temperature of 50°C. We also tested an alternative strategy for RNA extraction from FTA cards following the Whatman FTA Protocol BR01 (http://www.whatman.com/UserFiles/File/Protocols/Bioscience/BR01). Substantial costs for the recommended RNA processing buffer and our RNA yields severely compromised by the necessity for DNase digestion of extracted RNA prompted us to discontinue this approach.
(iii) RNAprotect***®*** cell reagent: RNA was extracted from 300 µl total volume (50µl whole blood plus 250µl RNAprotect reagent) using the RNeasy® plus mini kit protocol for spin column followed by on-column DNase digestion (all from Qiagen). All procedures followed those described in (i) with exception of the first step. This protocol starts with centrifugation for 15 min at 14’000 g. If a pellet was visible, all supernatant was removed and stored at -80°C for subsequent RNA extractions. If no pellet was visible, the centrifugation was repeated. If still no pellet was visible, 250 µl supernatant were removed and to the remaining 50 µl left in the tube RLT lysis buffer was added and RNA was extracted as described above in procedure (i).

The outcome in common of all our attempts to optimize RNA extraction points towards the use of on-column DNase digestion for minimizing loss of RNA.

#### DNA extraction

Parasite genomic DNA was extracted from all 311 blood samples, from 4 study participants these samples were missing. After removal of plasma and storage of blood pellets at -20°C for a maximum of two years, 50-150 µl blood pellet was used for DNA extraction (individual volumes were recorded) using FavorPrep™ 96-well genomic DNA extraction kit (Favorgen, Taiwan). DNA was eluted in 200 µl elution buffer and stored at -20°C.

### Molecular detection of *Plasmodium* parasites

**Figure 1:**
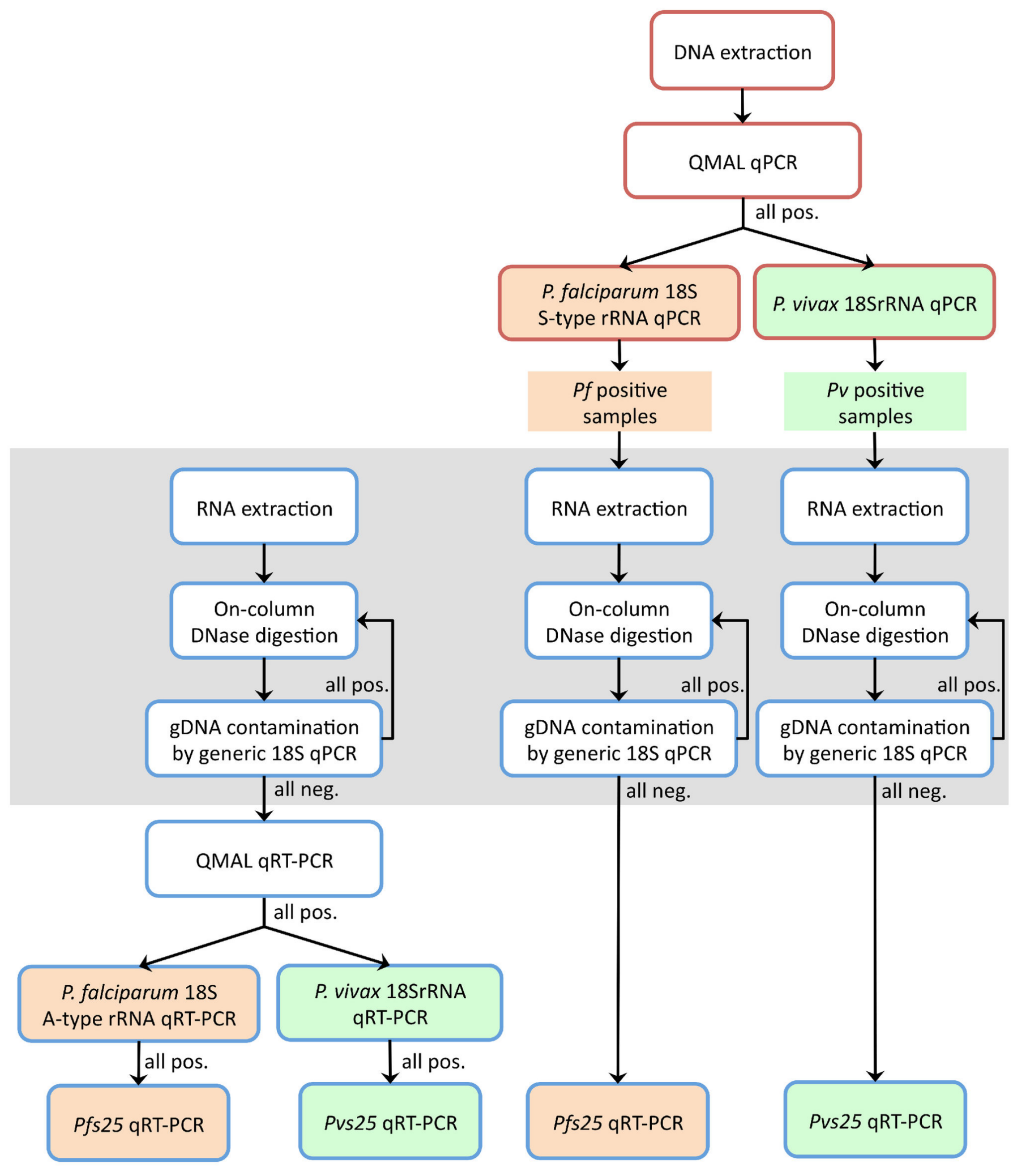
Flow diagram of molecular analyses performed for detection of asexual and sexual parasite stages of *P. vivax* and *P. falciparum* in field samples from PNG. Red and blue frames indicate assays done on DNA and on RNA, respectively. Orange and green boxes are *P. falciparum* and *P. vivax*-specific assays, respectively. *P. malariae* and *P. ovale* assays are not included in the diagram.

The workflow in Figure **1** depicts the series of consecutive assays performed with both RNA and DNA samples. The infecting Plasmodium species was determined by quantitative PCR (qPCR) using a gDNA template extracted from blood pellets and in parallel also by quantitative reverse-transcription PCR (qRT-PCR) using RNA obtained from the 3 different sampling methods.

#### RNA-based *Plasmodium* *species* diagnosis

After extraction all RNA samples were tested by qPCR targeting genes encoding 18S rRNA to confirm complete digestion of gDNA in a StepOne Plus® Real-Time PCR system (Applied Biosystems). This was followed by a generic qRT-PCR using the same generic primers and probe but on RNA in a one-tube reaction combining the reverse transcription and amplification reaction. Primers and probes are listed in Table **S1**, reaction mixes and PCR profiles are listed in Table **S2**. The RNA-based *P. falciparum* assay targeted A-type 18S rRNA transcripts expressed in asexual stages [17], whereas the generic assay targeting conserved regions would amplify all 5 copies of 18S rRNA genes in the genomes of *P. falciparum* and *P. vivax* [18]. All samples positive by the generic *Plasmodium* sp. assay were further analyzed by species–specific qRT-PCR reactions in a simplex reaction for *P. falciparum* and *P. ovale*, and as a duplex reaction for *P. vivax* and *P. malariae*. Primers and probes are listed in Table **S1**. Due to the generally higher parasitaemia in *P. falciparum* than in *P. vivax* infections, 18S rRNA transcripts in *P. falciparum* samples were highly abundant compared to 18S rRNA of for *P. vivax*. During extensive test evaluation we have observed a low level of aerosol-derived contamination when introducing negative controls, i.e. extraction of water. This low level of air-bourne contamination found in some, but not all negative samples, was corrected for by introducing a cut-off of 10 copies/µl extracted RNA for *P. falciparum* 18S rRNA qRT-PCR; for *P. vivax* no cut off was required. The cut off was identified in a plot of all measured transcript copy numbers by the point from which copy numbers rose above a steady baseline. By introducing a cut-off for *P. falciparum*, 51 previously *Pf* qRT-PCR positive samples were considered false positive. All except one sample had been *P. falciparum* negative by qPCR. This finding gave support to the choice of 10 copies/µl extracted RNA as our cut off.

Each plate carried a dilution series of assay-specific control plasmids with the respective template inserted at concentrations of 10^6^,10^4^ and 10^2^ copies of template/reaction in duplicates. For each plate standard curves were generated from these values for quantification of copy numbers in test samples.

#### DNA-based species diagnosis

As depicted in Figure **1**, all DNA samples were first tested for positivity for any *Plasmodium* parasite species by a generic assay. All samples positive by generic assay were quantified by simplex qPCRs for *P. falciparum* and *P. vivax. P. malariae* and *P. ovale* assays were not performed on DNA level, only on RNA level.

### Gametocyte-specific assays

For detection and quantification of *P. falciparum* and *P. vivax* gametocytes qRT-PCRs targeting the two orthologues *pfs25* and *pvs25* transcripts (GenBank accession no: AF193769.1 and GU256271.1, respectively) were developed and validated. These genes are the most frequently used markers for gametocyte detection in NASBA. *Pfs25* is highly expressed in mature gametocytes [4]. Sequences of oligonucleotides are given in Table **S1**, the composition of reaction mixes and thermo profiles are shown in Table **S2**. *Pfs25 and pvs25* primers as well as the *pfs25* HEX-BHQ1-labeled and *pvs25* FAM-BHQ1-labeled probes were selected within non-polymorphic positions identified by alignment of all publicly available nucleotide sequences. Of 138 *P. vivax pvs25* sequences, none showed polymorphism at sequences targeted by our assay, with the exception of a SNP observed in a single isolate, however, this DNA was no more available from the authors for confirmation of sequencing [19]. The region of *pfs25* targeted by our qRT-PCR assay is similar to that of the *pfs25* qNASBA assay of Schneider and coworkers [10], whose molecular beacon overlaps with the forward primer of our qRT-PCR assay. *Pvs25* primers and probe target the gene region also chosen by Beurskens and coworkers [11], whose molecular beacon is identical with our probe.

### Gametocyte trend line used for conversion of *pfs25* transcript copy numbers into gametocyte counts

A synchronized (5% sorbitol) 3D7 ring culture of 8% parasitaemia was induced to undergo gametocytogenesis at day -2 by reducing the hematocrit and doubling the Albumax concentration in the medium. At day -1, induced trophozoites were diluted to 2% and a second stress medium was added. At day 0, regular medium was added to the gametocytes (modified after [20]). At day 1 and until day 9, 50mM N-acetylglucosamine was added to the gametocytes to reduce surviving of asexual stages [21]. At day 12, gametocytes were purified by a percoll gradient [22] and counted in a Neubauer Cell Count Chamber at 3 different concentrations. The purified gametocytes were diluted in full medium to concentrations of 10’000, 1’000, 100, 50, 10, 5, 1, 0.5, 0.1, 0.05, 0.01, 0.005 gametocytes/µl in triplicates. 50 µl of gametocyte dilution was added to 250 µl RNAprotect cell reagent (Qiagen) and frozen at -20°C. RNA was extracted using the RNeasy plus 96well Kit (Qiagen) with an additional on column DNase digestion step and amplified on *pfs25* qRT-PCR and QMAL qPCR. A linear regression was applied on the log_10_ transformed copy numbers of the gametocyte trend line by R version 2.14.0 [23]. The regression coefficient (r^2^) was 0.95 (p<0.0001). The conversion from transcript copy numbers to gametocytes was as follows: gametocyte counts/µl whole blood = 10^-1.6225^ * (copy number pfs25 transcripts/µl whole blood) ^0.8518^.

### Light microscopy (LM)

Blood slides with thick and thin smears were collected in duplicate for each patient and examined microscopically for *Plasmodium* parasite density (asexual stages and gametocytes) and species identification. Three reads were done and densities counted over 200 white blood cells, which were then converted into parasite s/µl by assuming 8000 white blood cells/μl whole blood.

### Statistical analysis

Parasite counts by LM were multiplied by 40 (200WBC ◊ 8000WBC/µl blood) and log_10_ transformed. Template copy number/µl whole blood obtained from qPCR and qRT-PCR were log_10_ transformed. Only samples with positive cell counts in both tests were considered. For comparing molecular and microscopic parasite quantification methods, linear regression was applied to the log-transformed densities and DNA or transcript copy numbers. Correlation coefficients were calculated with R [23]. F-statistic was applied to test whether regressions (e.g., LM versus molecular detection) were significant.

## Results

### Validation of assays and Limit of Detection (LOD) for amplification of gDNA and cDNA

For each assay the respective PCR fragment was inserted into a plasmid as described previously [24]. Serial dilutions of these control plasmids were made in quintuplicates. LOD was defined as the lowest concentration of control plasmid (in copy number/μl) yielding positive results in >50% of parallel samples tested. LOD and amplification efficiencies (calculated as Efficiency = 10^(-1/Slope)^ -1) of each assay is listed in Table **S3**.

### Prevalence of *Plasmodium* *sp*. in study population by RNA-based versus DNA-based detection

Detection of any Plasmodium species (generic assay) as well as specific detection of *P. falciparum* or *P. vivax* was performed in parallel on DNA and RNA for all samples. DNA-based detection followed our previously described protocol [24]. For RNA-based detection by qRT-PCR the same primers and probes were utilized, except for the *P. falciparum* assay, which on RNA level targets the A-type 18S rRNA instead of the S-type gene as in qPCR [17]. The generic assays on RNA and DNA level were carried out on all samples, whereas the species-specific assays were only performed in samples previously positive by the generic assay according to Figure **1**. Due to the low local prevalence of *P. malariae* and *P. ovale*, both these species were only detected by RNA-based assays, DNA-based assays were omitted. To reduce complexity in Figure **1**, the performed *P. malariae* and *P. ovale* assays were not included.

The initial analysis, carried out to screen for the presence of any malaria parasite, detected 112/311 parasite positive DNA samples, whereas RNA-based 169/315 samples were positive. Such discrepancy in positivity mirrors the high sensitivity of detection when targeting highly abundant 18S rRNA transcripts (probably >10^6^ per cell), as opposed to only 5 copies of the 18S rRNA gene per genome [18].

All DNA or RNA samples positive by the generic assays were further analyzed by species specific qPCR or qRT-PCR assays, which also targeted 18S rRNA sequences, yet not the conserved part, but stretches instead that differed between *Plasmodium* species. We have compared parasite positivity obtained by both qPCR and qRT-PCR and by light microscopy in our samples (Table **1**). *P. falciparum* prevalence was 14.1% in DNA samples, but 24.1% in RNA samples. The discrepancy was even larger for *P. vivax*, with 19.6% DNA-based and twice as high RNA-based positivity (38.4%). As expected light microscopy provided the lowest prevalence rate (6.6% for *P. falciparum* and 13.3% for *P. vivax*). Because most parasite carriers were asymptomatic, many of these infections likely harbored low parasite densities around the detection limit of microscopy.

**Table 1:** Prevalence of asexual and sexual stages of *P. falciparum* and *P. vivax* detected by microscopy, qPCR or qRT-PCR in samples from 315 children from PNG.

	**Detection method of asexual *Plasmodium* stages**		
	**Light microscopy**	**DNA-based approach**	**RNA-based approach**
Sample size (N)	301	311	315
*P. falciparum* prevalence	20/301 (6.6%)	44/311 (14.1%)	76/315 (24.1%)
*Pf* gametocyte carriers in *Pf* pos.	7/20 (35.0%)	26/44 (59.1%)	31/76 (40.8%)
*P. vivax* prevalence	40/301 (13.3%)	61/311 (19.6%)	121/315 (38.4%)
*Pv g*ametocyte carriers in *Pv* pos.	23/40 (57.5%)	32/61 (52.4%)	46/121 (38.0%)

### Quantification of parasites

For most research questions quantitative parasitological data is desirable. When introducing molecular measures for parasite quantification, their performance with respect to the classical techniques needs to be evaluated. We therefore compared parasite counts by the different methods. Quantification of *P. vivax* was expected to be particularly difficult for two reasons: firstly, in our study area *P. vivax* densities are about 10 fold lower than *P. falciparum* densities [25], thus detection by PCR is more likely affected by the so called “Monte Carlo effect”, i.e., the random presence or absence of template in a tested DNA aliquot deriving from a blood sample of very low parasitaemia. The additional detection of a large number of scanty parasitaemias by the molecular assay of high sensitivity will lower the median parasite density compared to microscopy. Secondly, due to the presence of *P. vivax* schizont stages in the peripheral blood, a single parasite is not equivalent to one genome, but could account for a per parasite >20 fold higher copy number of the target gene.

**Figure 2:**
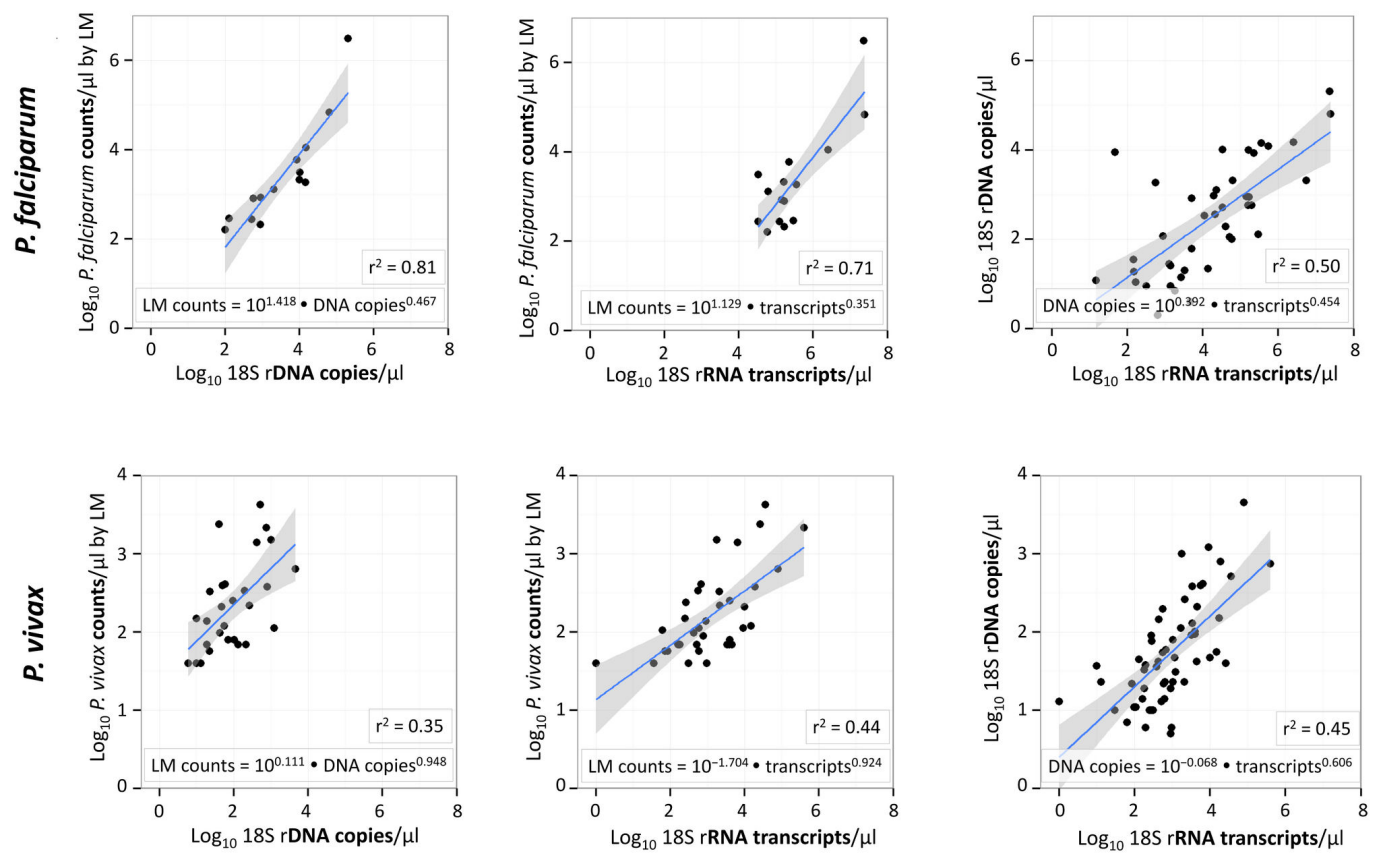
DNA- versus RNA-based quantification of *Plasmodium* parasites by qPCR and qRT-PCR of 18S rRNA genes or transcripts in comparison to light microscopy (LM). *P. falciparum* (upper panel) and *P. vivax* (lower panel). Boxed values indicate the correlation coefficient (r^2^) and the conversion functions extracted from these data. All correlation coefficients (r^2^) were significant (p-value < 0.001).

To permit parasite quantification based on target gene or transcript copy numbers detected by our 18S rRNA assays, we have plotted densities by light microscopy (LM) versus DNA- or RNA-based quantification (Figure **2**). Only samples positive by both compared tests were considered. For *P. falciparum* 17 LM/DNA, 20 LM/RNA and 42 DNA/RNA positive pairs were available, for *P. vivax* these were 29 LM/DNA, 37 LM/RNA and 58 DNA/RNA pairs. For *P. falciparum* (upper panel Figure **2**), parasite quantification by LM and qPCR correlated well (r^2^=0.81), when assuming presence of mainly ring/early trophozoite stage parasites in peripheral blood samples (equal to1 genome/parasite).

For *P. vivax* (lower panel Figure **2**) LM parasite counts and DNA copy numbers correlated less well (r^2^=0.35). Confidence intervals wider than those for *P. falciparum* denote a less robust quantification for *P. vivax*, likely due to the presence of schizonts or the overall lower densities. For both parasite species the correlation between the measurements for RNA transcripts versus gene copies was around 50% (Figure **2**, panels on the right). In Figure **2** all regressions on the log-transformed data were significant to a p-value less than 0.001.

Due to the lower sensitivity of LM compared to both molecular methods, only a very limited number of samples was available with data from all quantification methods, which does not represent a robust basis for conversion of molecular data. Nevertheless, we have explored this possibility for conversion and calculated the median parasite densities quantified by qPCR or qRT-PCR (Table **S4**) by using the algorithm determined in the regression analyses (shown in Figure **2**). As expected, mean parasite densities by DNA based quantification were lower than by LM, reflecting the contribution of the additional samples sub-patent by LM and with presumably lower parasite densities. RNA-based quantification was not consistent between *P. falciparum* and *P vivax*.

Our efforts to generate molecularly determined parasite counts provided preliminary evidence for a good predictive relationship between DNA copy numbers and microscopic parasite density, especially for *P. falciparum*. Yet, this approach needs further validation by a larger sample set to provide a solid mathematical function for this relationship.

### Co-infections with multiple *Plasmodium* *species*

To determine the overall prevalence of any *Plasmodium* *species* or of each specific species, we took into account all positive test results from DNA- and RNA-based assays (Table **S5**). Overall malaria parasite prevalence in the 315 children was 54.3%. Of these, almost a quarter had P. falciparum/P. vivax mixed infections (21.1%). Triple infections of *P. falciparum, P. vivax* and either *P. malariae* or *P. ovale* were seen in very few cases (0.6% and 3.5%). P. malariae single infections were less than 1%; no *P. ovale* single infection was observed.

### Gametocyte prevalence rates for *P. falciparum* and *P. vivax*

In our hands RNA extraction from FTA classic cards did not yield satisfactory results, despite efforts in optimizing the extraction protocol with the Qiagen RNeasy Plus mini kit. In contrast, gDNA could be extracted from these cards, but positivity in the *Plasmodium* species assays was reduced compared to results from the two alternative sampling methods (data not shown). Storage time >3 months or another, by us unnoticed problem during sampling, shipment or storage, all could have compromised RNA integrity on FTA cards. Our failure to detect gametocyte-specific RNA is in line with a similar work on field samples from Brazil [26].

**Figure 3:**
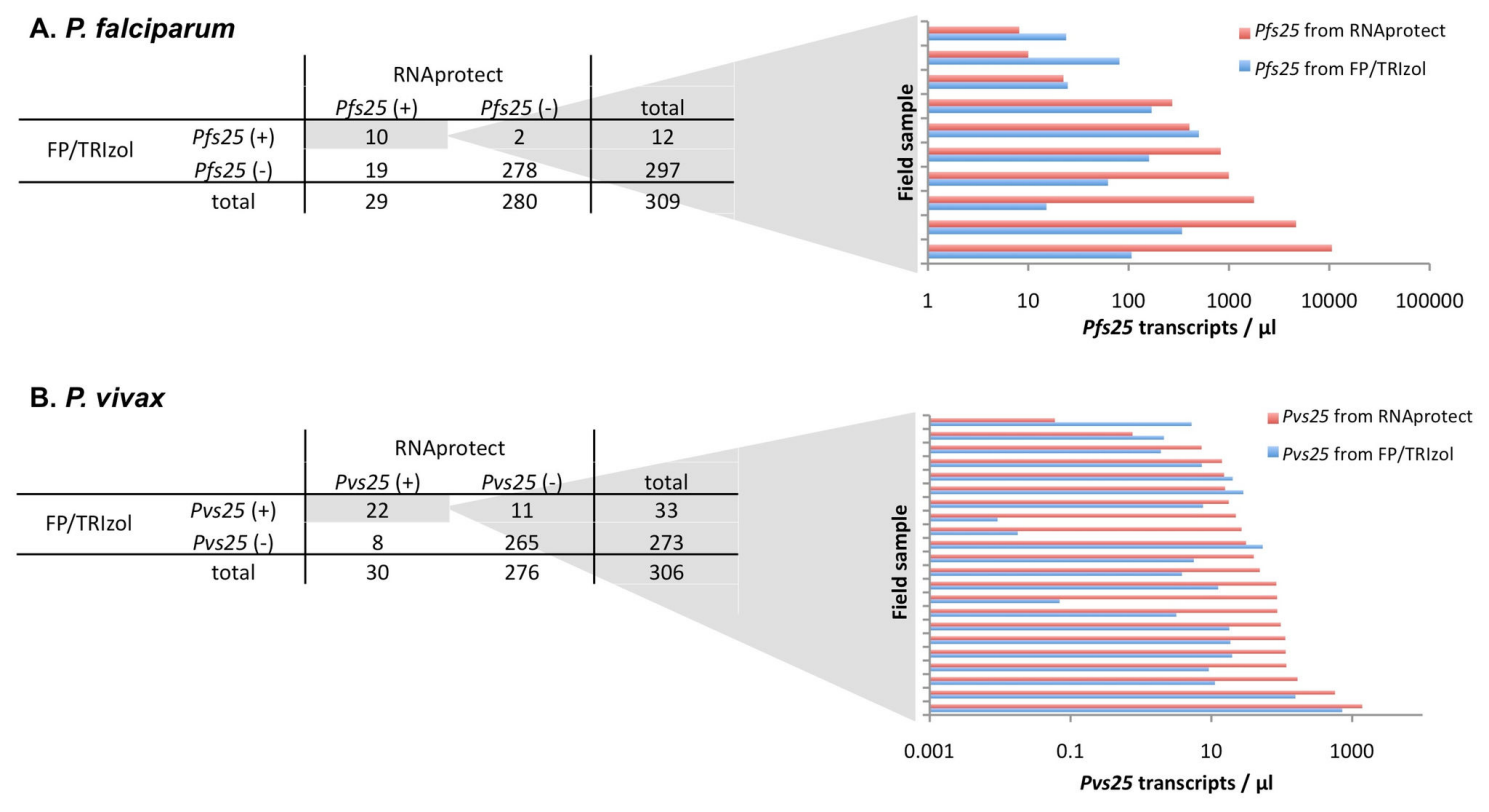
Comparison of two blood sampling strategies for measuring gametocyte prevalence rates. (A) *P. falciparum*, (B) *P. vivax*. Gametocyte positivity (left panel) and transcript copy numbers (right panel) are shown for RNAprotect solution versus filter paper soaked in TRIzol. Only samples were compared for which both measurements were available.

RNA was extracted successfully from all blood samples collected by both strategies, RNAprotect® and filterpaper/TRIzol®. Of 315 children tested by any sampling method, 32 and 46 carried *P. falciparum* and *P. vivax* gametocytes, respectively (Table **1**). To evaluate the differential performance of the two sampling approaches, we have compared the gametocyte positivity and transcripts numbers by either method for *P. falciparum* and *P. vivax* (Figure **3**). RNAprotect® sampling yielded more *P. falciparum* positive samples than filterpaper/TRIzol®. In samples positive by both methods, transcript copy numbers were higher for RNAprotect® sampling as shown in a comparison of paired results (Figure **3**, bar chart). For *P. vivax*, each of the methods missed about one third of gametocyte positive samples as compared to the summary result. The great fluctuation in *P. vivax* positivity and quantification is likely due to the overall lower density of *P. vivax* asexual stages and gametocytes. By LM no *P. falciparum* or *P. vivax* gametocytes were observed, thus confirming the well established superiority of molecular gametocytes detection. We have assessed how far gametocyte prevalence is associated with asexual densities (Figure **S1**). A positive association of high 18S rRNA transcripts and gametocyte prevalence was observed for *P. vivax* and *P. falciparum*. This association was also seen for 18S rDNA copy numbers of *P. vivax*, but not *P. falciparum*. The data available was rather limited; a more robust investigation of these relationships would require a larger sample set.

### Evaluation of *P. falciparum* and *P. vivax* gametocyte quantification assays

**Figure 4:**
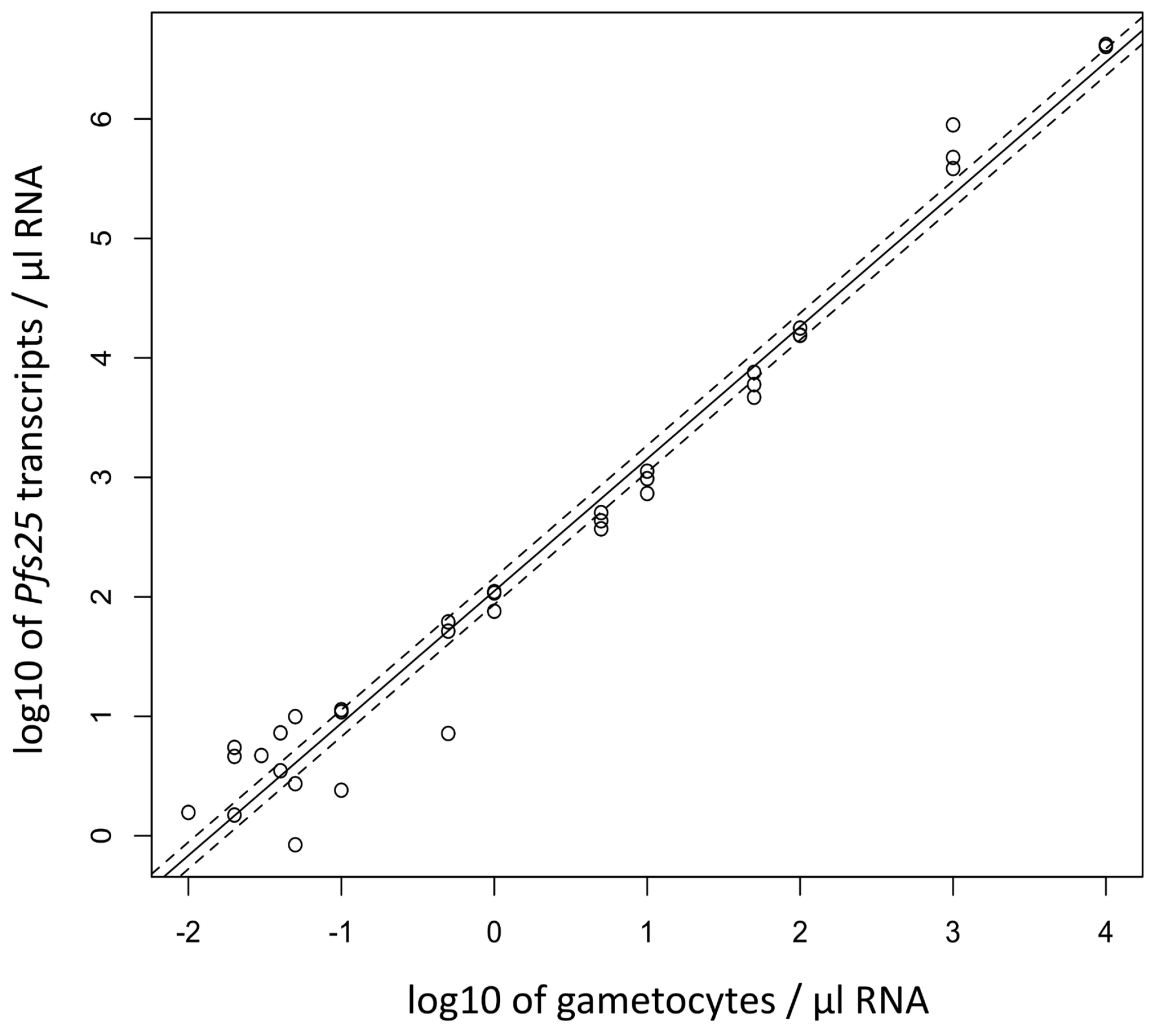
Gametocyte trend line generated with 3D7 *P. falciparum* in vitro culture for converting *pfs25* transcript copy numbers into gametocyte counts. Dashed lines indicate 95% confidence interval of intercept.

For quantification of gametocytes in a blood sample, the number of detected transcripts per sample is not meaningful without a standard curve that permits transforming copy numbers into gametocyte counts. Therefore a gametocyte trend line was generated from 3D7 in vitro culture by counting gametocytes prior to harvesting RNA. Figure **4** presents the standard curve used for estimating gametocyte loads in our samples and for establishing the detection limit of our qRT-PCR assays. According to the conversion factor obtained from regression analysis (see method section), one *P. falciparum* gametocyte corresponds to 87.05 *pfs25* transcript copies (95% CI: 65.55-115.60). This translates into a limit of detection (LOD) of 0.02-0.05 gametocyte / µl blood when 50 µl blood or parasite culture was subject to RNA extraction. If volumes larger than 50 µl of blood would be sampled, our *pfs25* qRT-PCR would even permit an almost 2-fold greater sensitivity (1 gametocyte / 100 µl blood). This example indicates that LOD based on transcript copy numbers rather than gametocytes only describes detection potential, whereas presence or absence of gametocytes determines the effective sensitivity. Our LOD compares to that published for qNASBA-based gametocyte detection [27]. Due to the lack of *P. vivax in vitro* culture, we had to use the *P. falciparum* based conversion factor for calculating *P. vivax* gametocyte loads. Median gametocyte numbers per µl blood were 0.99 (1^st^ quartile, 3^rd^ quartile: 0.27, 4.95) for *P. falciparum* and 0.34 (1^st^ quartile, 3^rd^ quartile: 0.11, 0.68) for *P. vivax*. So far we failed to establish gametocyte assays for *P. malariae* and *P. ovale* due to yet little success to find *pfs25* orthologues in these species.

### Effect of storage duration on stability of *pfs25* transcripts

**Figure 5:**
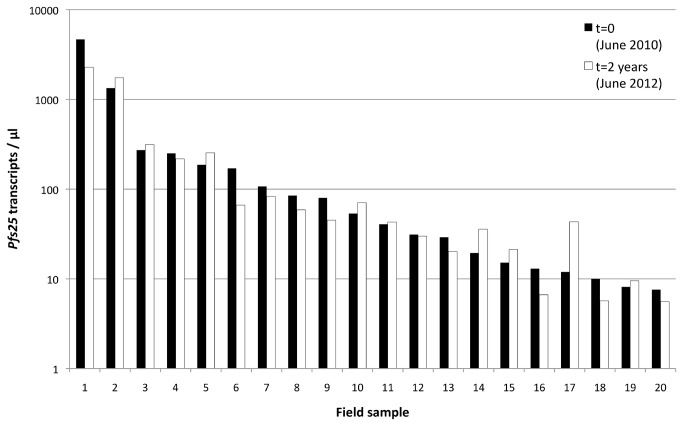
Effect of extended storage time on *pfs25* transcripts. 20 samples were chosen to represent a wide range of transcript copy numbers at start of the 2 yr storage period. The initial copy numbers (black bars) are shown next to copy number detected 2 yrs later in the same RNA sample (white bars).

Two years after extraction of RNA from both types of samples, RNAprotect® and filter paper/TRIzol®, we have repeated *pfs25* qRT-PCR of a subset of all samples representing the full range of transcript copy numbers. Sample pairs plotted side by side did not indicate compromised RNA stability after 2 years of storage at -80°C (Figure **5**). Other protein coding transcripts were not tested and the stability of *pfs25* RNA may represent an exception rather than the rule.

## Discussion

The difference in DNA- versus RNA-based *Plasmodium* species determination derives from the dramatic difference in the number of templates per parasite. Each *Plasmodium* parasite harbors only 5 copies of the *18S rRNA* gene, 3 S-type (detected by our qPCR assay) and 2 A-type genes [18], whereas many thousands or even some million copies of *18S rRNA* transcripts can be expected per cell. The use of these extremely abundant transcripts for parasite detection warrants great care during RNA extraction, a large number of negative controls and precise definition of a cut-off to avoid false positives through potential aerosols (low level of signal caused by airborne templates). Depending on the research question, outermost sensitivity may be desired, e.g. when searching for very rare infections in a close to elimination setting. When pooling samples for massive parasitological screening, such high sensitivity is a prerequisite. These issues might attract further attention in the eliminations context when sensitive diagnosis is required for detection of asymptomatic parasite carriers with low density, as well as for tracking both asexual and sexual stages in parallel [26].

Sensitivity of detection of blood-borne parasites is defined in a major way by the volume of blood analyzed, which for very low parasitaemias may or may not contain a parasite by chance. In asymptomatic infections the probability of detection of malaria parasites is hampered by generally low densities. Accordingly, the chances of gametocyte detection are even more limited. Our gametocyte assay was found to be sufficiently sensitive to reproducibly detect a single gametocyte in 50 µl whole blood. Because 1 gametocyte corresponded on average to 87 *pfs25* transcript copies, it seems likely that extracting RNA from 100 µl whole blood could have improved detection of scarce gametocytaemia. Generally the blood volume collected in population studies is limited, as up to 250 µl of whole blood can be obtained by finger prick, the usual sampling method for large-scale field surveys and cohort studies. After setting aside aliquots for blood films, serology and DNA extraction, starting material for gametocyte detection is restricted, but should be maximized according to the above results.

When describing DNA- or RNA-based diagnostic assays, the sensitivity of parasite detection is generally presented on the basis of experiments with *in vitro* cultured parasites under optimal laboratory conditions [28]. Yet, for *P. vixax* *in vitro* culture is not available. The approach used in our study takes into account the conditions under which field samples were collected and shipped, mirroring the setting of routine malariological surveillance. To permit direct comparison of *P. falciparum* and *P. vivax* densities, parasites of both species ideally should be quantified in the same way by extracting an algorithm for converting template copy numbers detected in each sample into parasite s/µl whole blood. We have determined this relationship (Figure **2**) and quantified the median parasite densities for *P. falciparum* and *P. vivax* based on the correlations observed (Table **S4**). For both species DNA based quantification produced mean parasite densities lower that those by LM, because the increased molecular detection contributed primarily samples with parasite densities below the detection limit of LM. RNA-based parasite densities revealed a much greater variance than DNA-based quantification and thus provided less precise estimates of parasite density. This likely reflects small variations in sampling or processing, or could derive from longer delays in reaching the molecular laboratory for some of the samples. The generally adverse and variable field conditions could differentially impair the RNA quality in certain samples. Our data suggests that quantification based on RNA needs further validation.

Our study aimed at improving blood-sampling techniques in the field at remote sites. For this we have optimized gametocyte detection and quantification and compared RNA stability achieved by three sampling methods. We were only able to extract RNA reliably from samples stored in TRIzol and RNAprotect. Samples stored on FTA cards did not give satisfactory results. In contrast, Pritsch and co-workers [12] reported successful RNA extraction from Whatman FTA classic cards. The comparability of these sampling procedures with our results is limited, because our starting material consisted of low density asymptomatic field samples that had been stored for 6 months, whereas storage time was not specified in the earlier publication [12]. In a comprehensive comparison of several filter papers for collecting low density gametocytes, Jones and coworkers [8] reported a much lower amplification success of pfs25 transcripts from FTA classic cards compared to Whatman 3MM untreated filter paper. This is in line with our observations. But also in that study filter papers were stored only for up to 3 months. In our experience, samples from major field surveys very often are stored for periods longer than 3 months and longer than originally anticipated. Storage periods of 4 weeks of 3 months, which is normally evaluated in comparative analysis of sampling materials, is very brief given the average duration of large scale field studies. Particularly cohort studies may well last over a year, e.g. to capture seasonal changes. We therefore reported the sensitivity of asexual stage and gametocyte detection after extended storage periods of up to 1 year and confirmed the stability of *pfs25* transcripts even after long term storage of 2 years.

Whatman 3MM non-impregnated filter paper has been used successfully in several recent studies [7,12,29], all reporting good RNA yields from blood spotted on filter paper without addition of any RNA-stabilizing reagent such as RNAprotect or TRIzol, but storage duration in these studies was up to 3 months only. We have shown that even long term storage of the blood impregnated 3MM filter paper in TRIzol is possible.

RNAprotect sampling showed best results, despite a delay of several hours until whole blood samples were transferred from EDTA microtainer into RNAprotect reagent. This contrasts with conclusions of a previous study that suggested compromised RNA stability after a 6 hrs delay prior to addition of RNAprotect [7]. The optimal sampling of whole blood in a RNA stabilizing agent would involve blood collection directly into tubes containing RNAprotect. But skin contact to RNAprotect, an irritant substance, should be avoided; thus finger prick blood is first collected in microtainers prior to transferring a 50 µl or larger aliquot into a tube pre-filled with 5 volumes RNAprotect solution. In this field survey we attempted to limit the delay until transfer in stabilizing agent to a maximum of 4 hours. A recent major field study conducted in Burkina Faso has demonstrated the feasibility of mixing whole blood with RNAprotect reagent directly at the site of blood collection without any delay [30]. In conclusion, for field settings far away from laboratory facilities, the latter approach of transferring whole blood directly into RNAprotect immediately after blood collection represents the optimal strategy, which, however, requires thorough training of field staff, e.g. on contamination-free pipetting of an aliquot whole blood from a microtainer into the prepared RNAprotect tube.

By detecting transcripts from late stage V gametocytes, we targeted specifically the parasite population in the human host most relevant for transmission. This depicts the impact of transmission-reducing interventions more closely than markers of earlier gametocyte stages, as not all committed rings might successfully develop into mature gametocytes. The presence of gametocytes is no evidence for subsequent transmission success of gametocytes to the vector. The relationship of gametocyte densities and successful infection of mosquitoes is of great relevance for molecular monitoring of interventions, but only recently first results were published on the prediction of mosquito infection from gametocyte densities [3]. It remains to be shown how molecular gametocyte counts in the host compare to the classical measure of transmission, the entomological inoculation rate [31]. We have evaluated procedures for gametocyte detection and quantification. Sampling strategy and molecular assays can be considered robust tools for molecular epidemiological studies and might prove valuable for estimating the impact of transmission-reducing interventions, such as drugs, vaccines, or vector control.

## Supporting Information

Figure S1 **Proportion of gametocyte carriers (gray) among *P. falciparum* (upper panel) and *P. vivax* (lower panel) infections separated into three copy number categories for 18S rDNA detected by qPCR or 18S rRNA transcripts detected by qRT-PCR.** Sample size of both groups within a category is indicated by numbers within the bars.
(EPS)

Table S1 **Primer and probe sequences.**
(DOC)

Table S2 **PCR profiles and reaction mixes.**
(DOC)

Table S3 **Limit of detection and amplification efficiencies of all molecular markers determined with control plasmids.**
(DOC)

Table S4 **Median density of *P. falciparum* and *P. vivax* parasite s/µl detected by microscopy, qPCR or qRT-PCR in samples from 315 children from PNG.**
(DOC)

Table S5 **Overall parasite prevalence derived from combined results of DNA- and RNA-based detection methods in study population (n=315) and distribution of mixed species co-infections in parasite positive samples.**
(DOC)
