# Supplementary material for: Strategies for Detection of Plasmodium species Gametocytes
Source: PLoS One. 2013 Sep 27;8(9):e76316. doi: 10.1371/journal.pone.0076316 (PMC3848260; doi:10.1371/journal.pone.0076316)
Supplement: Table S1 — Primer and probe sequences. (DOC) [file pone.0076316.s002.doc]

**Supplementary Table S1**. Primer and probe sequences.

| **A. Generic qPCR and qRT-PCR assays** (target: conserved regions in 18S rRNA) | | |
| --- | --- | --- |
| **Species** | **Primer** | **Sequence (5’ -> 3’)** |
| ***Plasmodium sp.*** | **QMAL_fw** | TTA GAT TGC TTC CTT CAG TRC CTT ATG* |
|  | **QMAL_rev** | TGT TGA GTC AAA TTA AGC CGC AA |
|  | **QMAL_probe** | **FAM-**TCA ATT CTT TTA ACT TTC TCG CTT GCG CGA **–BHQ** |
|  | | |
| **B. Species-specific qPCRs and qRT-PCR assays** | | |
| **Species** | **Primer** | **Sequence (5’ -> 3’)** |
| ***P. falciparum* (DNA)** | **Pf_S18S_fw** | TAT TGC TTT TGA GAG GTT TTG TTA CTT TG |
|  | **Pf_S18S_rev** | ACC TCT GAC ATC TGA ATA CGA ATG C |
|  | **Pf_S18S_probe** | FAM-ACG GGT AGT CAT GAT TGA GTT-MGB-BHQ |
| ***P. falciparum* (RNA)** | **Pf_A18S_fw** | TCC GAT AAC GAA CGA GAT CTT AAC |
|  | **Pf_A18S_rev** | ATG TAT AGT TAC CTA TGT TCA ATT TCA |
|  | **PF_A18S_probe** | FAM-TAG CGG CGA GTA CAC TAT A-MGB-BHQ |
| ***P. vivax* (DNA & RNA)** | **Pv_18S_fw** | GCT TTG TAA TTG GAA TGA TGG GAA T |
|  | **Pv_18S_rev** | ATG CGC ACA AAG TCG ATA CGA AG |
|  | **Pv_18S_probe** | HEX-AGC AAC GCT TCT AGC TTA -MGB-BHQ |
| ***P. malariae* (DNA & RNA)** | same primers and probe as in ref. 24 | |
| ***P. ovale* (DNA & RNA)** | same primers and probe as in ref. 24 | |
|  | | |
| **C. Gametocyte-specific *pfs 25* and *pvs25* qRT-PCR** | | |
| **Species** | **Primer** | **Sequence (5’>3’)** |
| ***P. falciparum*** | **pfs25_fw** | GAA ATC CCG TTT CAT ACG CTT G |
|  | **pfs25_rev** | AGT TTT AAC AGG ATT GCT TGT ATC TAA |
|  | **pfs25_probe** | HEX-TGT AAG AAT GTA ACT TGT GGT AAC GGT-BHQ1 |
| ***P. vivax*** | **pvs25_fw** | ACA CTT GTG TGC TTG ATG TAT GTC |
|  | **pvs25_rev** | ACT TTG CCA ATA GCA CAT GAG CAA |
|  | **pvs25_probe** | FAM-TGC ATT GTT GAG TAC CTC TCG GAA-BHQ1 |

* wobble R = A/G
