# Supplementary material for: Strategies for Detection of Plasmodium species Gametocytes
Source: PLoS One. 2013 Sep 27;8(9):e76316. doi: 10.1371/journal.pone.0076316 (PMC3848260; doi:10.1371/journal.pone.0076316)
Supplement: Table S2 — PCR profiles and reaction mixes. (DOC) [file pone.0076316.s003.doc]

**Supplementary Table S2**. PCR profiles and reaction mixes.

| **A. qPCR** | | | |
| --- | --- | --- | --- |
| **qPCR Reaction mix**^1^ | | | |
| Total volume  12 μL | 1X gene expression master mix^2^ | | |
|  | 800 nM primer mix | | |
|  | 200 nM probe^2^ | | |
|  | 2µl of RNA | | |
|  | | | |
| **qPCR Thermo profile**^3^ | | | |
| **Stage** | **Step** | **Temperature** | **Time** |
| Holding | UDG | 50°C | 2 minutes |
| Holding | Activation of AmpliTaq polymerase | 95°C | 10 minutes |
| Cycling (45x) | Denature | 95°C | 15 seconds |
|  | Anneal/Extend | 58°C | 1 minute |
|  | | | |
| **B. qRT-PCR**: one-tube protocol using the TaqMan® RNA-to-C_T_™ *1-Step* Kit^2^ | | | |
| **qRT-PCR Reaction mix** ^1^ | | | |
| Total volume 12.5 μL | 1X RT-to-CT master mix^2^ | | |
|  | 800 nM primer mix | | |
|  | 200 nM probe^2^ | | |
|  | 2µl of RNA | | |
|  | 0.3 µl of Taqman RT enzyme mix (ArrayScript™ UP Reverse Transcriptase and RNase Inhibitor) | | |
|  | | | |
| **qRT-PCR Thermo profile**^3^ | | | |
| **Stage** | **Step** | **Temperature** | **Time** |
| Holding | Reverse transcription | 48°C | 15 minutes |
| Holding | Activation of AmpliTaq polymerase | 95°C | 10 minutes |
| Cycling (45x) | Denature | 95°C | 15 seconds |
|  | Anneal/Extend | 58°C | 1 minute |

^1^ Reaction mix was prepared on a template-free bench wiped with 2.5M hypochlorite solution. Prepared master mix was added to the reaction plate before transfer to PCR cabinet for template addition. Applied Biosystem’s MicroAmp® 0.1ml Fast Optical 96-Well Reaction Plate was used for both qPCR and qRT-PCR.

^2^ Life Technologies Applied Biosystems, Zug, Switzerland

^3^The GENEX standard thermo profile of StepOnePlus Real-Time PCR system (Applied Biosystems) was modified for both qPCR and qRT-PCR. A maximum of 45 cycles of amplification was set. And all samples with Ct value ≤45 were considered positive.
