# Supplementary material for: Strategies for Detection of Plasmodium species Gametocytes
Source: PLoS One. 2013 Sep 27;8(9):e76316. doi: 10.1371/journal.pone.0076316 (PMC3848260; doi:10.1371/journal.pone.0076316)
Supplement: Table S3 — Limit of detection and amplification efficiencies of all molecular markers determined with control plasmids. (DOC) [file pone.0076316.s004.doc]

**Supplementary Table S3**. Limit of detection and amplification efficiencies of all molecular markers determined with control plasmids.

| **Assay** | **Limit of detection** copy number /μl^*^ | **Amplification efficiencies** |
| --- | --- | --- |
| Generic 18S rRNA | 1 | 96.5 |
| *P. falciparum* 18S rRNA (S-type) | 1 | 82.5 |
| *P. vivax* 18S rRNA | 3 | 82.2 |
| *P. malariae* 18S rRNA | 1 | 99.9 |
| *P.ovale* 18S rRNA | 1 | 92.8 |
| *pfs25* | 1 | 95.2 |
| *pvs25* | 0.5 | 92.0 |

# ^*^determined by serial dilution in quintuplicate of control plasmids (PCR template = insert of control plasmid).
