# Supplementary material for: Strategies for Detection of Plasmodium species Gametocytes
Source: PLoS One. 2013 Sep 27;8(9):e76316. doi: 10.1371/journal.pone.0076316 (PMC3848260; doi:10.1371/journal.pone.0076316)
Supplement: Table S4 — Median density of P. falciparum and P. vivax parasite s/µl detected by microscopy, qPCR or qRT-PCR in samples from 315 children from PNG. (DOC) [file pone.0076316.s005.doc]

**Supplementary Table S4**. Median density of *P. falciparum* and *P. vivax* parasites/μl detected by microscopy, qPCR or qRT-PCR in samples from 315 children from PNG.

|  | **Quantification method of asexual *Plasmodium* stages** | | |
| --- | --- | --- | --- |
|  | **Light microscopy** | **DNA-based approach** | **RNA-based approach** |
| ***P. falciparum* quantification** median [ 1st quartile, 3rd quartile ] | 1071 [ 285, 3839] | 350 [ 24, 1898 ] | 1297 [ 164, 36904 ] |
| ***P. vivax* quantification** median [ 1st quartile, 3rd quartile ] | 106 [ 69, 330 ] | 8 [ 6, 13 ] | 98 [ 66, 160 ] |
