# Supplementary material for: Strategies for Detection of Plasmodium species Gametocytes
Source: PLoS One. 2013 Sep 27;8(9):e76316. doi: 10.1371/journal.pone.0076316 (PMC3848260; doi:10.1371/journal.pone.0076316)
Supplement: Table S5 — Overall parasite prevalence derived from combined results of DNA- and RNA-based detection methods in study population (n=315) and distribution of mixed species co-infections in parasite positive samples. (DOC) [file pone.0076316.s006.doc]

**Supplementary Table S5.** Overall parasite prevalence derived from combined results of DNA- and RNA-based detection methods in study population (n=315) and distribution of mixed species co-infections in parasite positive samples.

| **Assay / marker gene** | **Positivity**^1^ |
| --- | --- |
| *Plasmodium* generic assay | 171/315 (54.3%) |
| *Pf* single infection | 32/171 (18.7%) |
| *Pv* single infection | 79/171 (46.2%) |
| *Pm* single infection | 1/171 (0.6%) |
| *Po* single infection | 0/171 (0%) |
| *Pf + Pv* infection | 36/171 (21.1%) |
| *Pf + Pm* infection | 3/171 (1.8%) |
| *Pf* + *Po* infection | 0/171 (0%) |
| *Pv + Pm* infection | 2/171 (1.2%) |
| *Pv + Po* infection | 0/171 (0%) |
| *Pf + Pv + Pm* infection | 1/171 (0.6%) |
| *Pf + Pv + Po* infection | 6/171 (3.5%) |
| *Pf + Pv + Pm + Po* infection | 0/171 (0%) |
| missed species typing^2^ | 11/171 (6.4%) |
| **any *Pf*** | 78/171 (45.6%) |
| **any *Pv*** | 124/171 (72.5%) |
| **any *Pm*** | 7/171 (4.1%) |
| **any *Po*** | 6/171 (3.5%) |

^1^The slight discrepancy to those prevalence rates given in Table 1 derives from very few samples positive by DNA-based detection, but negative by RNA-based detection. Accordingly, the summary result given here shows a slightly higher positivity.

^2^Eleven samples were positive for the *Plasmodium* genus-specific assay, but were negative in all species-specific assays. All but one of these 11 samples derived from RNA-based detection and were characterized by very low copy number (<10 transcripts). These samples must be considered false positive. Due to highly abundant 18S rRNA transcripts in each cell, a low level of aerosol derived contamination is possible. In principle, this issues can be addressed by introducing a cut-off (e.g. for *P. falciparum* 18S rRNA qRT-PCR we in fact applied a cut-off of 10 copies/µl extracted RNA). But our use of the generic assay for screening for all parasite species did not permit application of a stringent cut-off, which according to the occurrence of very high parasite densities would be oriented at *P. falciparum*. Cut off application to *P. vivax* would lead to exclusion of some very low density infections. In consequence, the true parasite prevalence by our generic assay most probably is slightly lower, i.e. excluding the 11 potentially false positive samples and thus amounting to 160/315 (50.8%).
